# Personality Disorder and Physical Health Comorbidities: A Link With Bone Health?

**DOI:** 10.3389/fpsyt.2020.602342

**Published:** 2020-12-08

**Authors:** Lana J. Williams, Shae E. Quirk, Heli Koivumaa-Honkanen, Risto Honkanen, Julie A. Pasco, Amanda L. Stuart, Bianca E. Kavanagh, Jeremi Heikkinen, Michael Berk

**Affiliations:** ^1^Deakin University, Institute for Mental and Physical Health and Clinical Translation, School of Medicine, Geelong, VIC, Australia; ^2^University Hospital Geelong, Barwon Health, Geelong, VIC, Australia; ^3^Institute of Clinical Medicine/Psychiatry, University of Eastern Finland, Kuopio, Finland; ^4^Kuopio Musculoskeletal Research Unit, Institute of Clinical Medicine, University of Eastern Finland, Kuopio, Finland; ^5^Departments of Psychiatry, South-Savonia Hospital District, Mikkeli, Finland; ^6^Departments of Psychiatry, North Karelia Central Hospital, Joensuu, Finland; ^7^Departments of Psychiatry, SOTE, Iisalmi, Finland; ^8^Department of Psychiatry, Oulu University Hospital, Oulu, Finland; ^9^Department of Medicine-Western Health, The University of Melbourne, St. Albans, VIC, Australia; ^10^Department of Psychiatry, The University of Melbourne, Parkville, VIC, Australia; ^11^Orygen the National Center of Excellence in Youth Mental Health, Center for Youth Mental Health, Florey Institute of Neuroscience and Mental Health, University of Melbourne, Parkville, VIC, Australia

**Keywords:** comorbidity, personality disorder, psychiatry, physical health, medical condition, bone

## Abstract

We examined whether personality disorders (PDs) (any, cluster A/B/C) were associated with bone mineral density (BMD) in a population-based sample of Australian women (*n* = 696). Personality and mood disorders were assessed using semi-structured diagnostic interviews. BMD was measured at the spine, hip, and total body using dual-energy x-ray absorptiometry (GE-Lunar Prodigy). Anthropometrics, medication use, physical conditions, and lifestyle factors were documented. The association between PDs (any, cluster A/B/C) and BMD (spine/hip/total body) was examined with multiple linear regression models. The best models were identified by backward elimination including age, weight, physical activity, smoking status, alcohol consumption, dietary calcium intake, mood disorders, physical multimorbidity, socioeconomic status, and medications affecting bone. The variables were retained in the model if *p* < 0.05. All potential interactions in final models were tested. Those with cluster A PD, compared to those without, had 6.7% lower hip BMD [age, weight adjusted mean 0.853 (95% CI 0.803–0.903) vs. 0.910 (95% CI 0.901–0.919) g/cm^2^, *p* = 0.027] and 3.4% lower total body BMD [age, weight, smoking, alcohol, calcium adjusted mean 1.102 (95% CI 1.064–1.140) vs. 1.139 (95% CI 1.128–1.150) g/cm^2^, *p* = 0.056]. No associations were observed between cluster B/C PDs and hip/total body BMD or between any of the PD clusters and spine BMD. To our knowledge, this study is the first to investigate the bone health of women with PD in a population-based sample. Given the paucity of literature, replication and longitudinal research including the examination of underlying mechanisms and sex differences are warranted.

## Introduction

Emerging early in the life span, a personality disorder (PD) presents as enduring patterns of maladaptive thinking, emotional and inner experiences, and behaviors—causing significant distress and impairment (1). PDs have traditionally been conceptualized as 10 distinct disorders that are organized within three clusters: A, “odd-eccentric” features; B, “dramatic/emotional/erratic” features; and C, “anxious/fearful” features (1).

Growing evidence points to adverse physical health comorbidities among people with PD, including chronic physical, pain, and sleep conditions (2, 3)—however, little is known regarding the factors underlying these associations (2). PD also appears to be associated with musculoskeletal problems (2, 3); however, there is a paucity of research examining the underlying bone health of people with PD.

Psychological/behavioral factors including impulsivity and self-sabotaging type behaviors, which are typical of “cluster B” symptomatology, appear to lead to difficulties with general health treatment compliance (4) and thus plausibly influence the physical health of people with these PDs. Biological/other health-related factors are suggested to include (but may not be limited to) multiple and long-term poor health-related lifestyle choices and medication use (5, 6) and metabolic syndrome (3). However, little is known whether different PD clusters (i.e., different presentations of PD symptomatology) are independently associated with poorer bone health compared to people without PDs.

Kahl et al. (7, 8) conducted the first clinical-based studies showing that comorbid borderline PD (a cluster B PD) and depressive disorders were associated with reduced bone mineral density (BMD) in younger women. Yet, there are no studies from the population-based setting or across the wider adult age range. Therefore, we explored direct associations between PD and BMD in a large population-based sample of Australian women—exploring the role of age, anthropometrics, lifestyle factors, multimorbidity, medication use, and mood disorders.

## Materials and Methods

This study examined data from women participating in the population-based Geelong Osteoporosis Study (GOS) in Australia (9). Originally, 1,494 women (response rate 77.1%) were randomly selected from the electoral rolls for the Barwon Statistical Division during 1994–1996. Complete details have been published elsewhere (9–11). We utilized data collected from participants who returned for assessment during 2011–2014. Participants who did not complete a psychiatric assessment (*n* = 80), had missing BMD data (*n* = 50), or with multiple PD clusters (*n* = 22) were excluded. This study consisted of *n* = 696 women aged ≥28 years. Barwon Health Human Research Ethics Committee approved the study, and written informed consent was obtained from all participants.

Areal BMD (g/cm^2^) was measured at the posterior–anterior (PA) spine (L2–4), femoral neck (hip), and total body including head using dual-energy X-ray absorptiometry (Prodigy; GE Lunar, Madison, WI, USA). Technicians carried out all examinations and performed daily calibrations of the densitometer with equipment-specific phantoms. Osteoporosis was determined by a BMD T-score of <-2.5 at the spine and/or hip (12).

The Structured Clinical Interview for Diagnostic and Statistical Manual of Mental Disorders (DSM) Axis I Disorders, non-patient edition (SCID-I/NP) identified lifetime mood disorders [i.e., major depressive disorder (MDD), minor depression, bipolar disorder, dysthymia, mood disorders due to a general medical condition, and substance-induced mood disorder] (13). The Structured Clinical Interview for DSM-IV Axis II personality disorders (SCID-II) identified PDs (14). Consistent with the Diagnostic and Statistical Manual of Mental Disorders (DSM-5), the 10 PDs were categorized into three clusters: cluster A PDs (i.e., schizoid, paranoid, and schizotypal PDs); cluster B PDs (i.e., antisocial, borderline, narcissistic, and histrionic PDs); and cluster C PDs (i.e., avoidant, dependent, and obsessive–compulsive PDs) (1). As previously published, all interviews were conducted by trained personnel with post-graduate qualifications in psychology (10).

Weight and height were measured to the nearest 0.1 kg or 0.1 cm, respectively. Current smoking and mobility were self-reported. Participants were deemed active if vigorous or light exercise was performed regularly; otherwise, participants were classified as sedentary. Alcohol consumption and calcium intake were estimated from a validated food frequency questionnaire (15). Current medication use affecting bone was recorded, including antiresorptive agents (bisphosphonates/selective estrogen receptor modulators), oral hormone replacement therapy, oral glucocorticoids, and antidepressants. Participants brought lists of medications or containers to the assessment to ensure accurate recordings. Participants self-reported the presence of physical conditions. These were confirmed by medical record, medication use, or clinical data, where possible, and included arthritis, cardiovascular disease, thyroid disorders, metabolic disorders, gastrointestinal disease, gastroesophageal reflux disease, syncope and seizures, recurrent headaches, pulmonary diseases, psoriasis, liver diseases, and cancers (16). Utilizing these data, we defined physical multimorbidity as the presence of ≥5 lifetime physical health disorders (17, 18). Area-based socioeconomic status (SES) was determined using the Index of Relative Socio-economic Advantage and Disadvantage (IRSAD). A lower score (Quintile 1) indicated greater disadvantage; a higher score, greater advantage (Quintile 5) (9).

Statistical analyses were performed using Minitab (version 18). Group differences were determined using *t*-tests for parametric variables, Kruskal–Wallis for nonparametric continuous variables—for discrete variables, chi-square tests or Fisher's exact test when expected cell counts were less than five. The association between PD (any, cluster A/B/C) and BMD (spine/hip/total body) was examined with multiple linear regression in unadjusted models (Model I) and “best models” (Model II). The best models were identified by backward elimination including age, weight, physical activity, smoking status, alcohol consumption, dietary calcium intake, mood disorders, physical multimorbidity, SES, and medications affecting bone (*c.f*. above). The variables were retained in the model if *p* < 0.05. All potential interactions in final models were tested.

## Results

Of 696 women, 131 (18.8%) met the criteria for a PD. Women with PD were more likely to be younger, heavier, sedentary, use antidepressants, had a lifetime mood disorder, and had higher hip and total body BMD than those without PD (all *p* < 0.05); otherwise, the groups were similar (Table 1).

**Table 1:** Characteristics of all and those with and without a PD.

	**All**	**PD**	**No PD**	p^*^
	***n* = 696**	***n* = 131**	***n* = 565**	
Age (years)	56.2 (42.6–67.6)	52.2 (38.7–63.8)	57.1 (43.3–68.8)	0.012
Weight (kg)	71.5 (62.2–83.8)	77.0 (65.6–87.9)	70.1 (61.9–82.6)	0.003
Height (m)	1.62 ± 0.06	1.63 ± 0.07	1.62 ± 0.06	0.274
Smoking (current)	78 (11.4%)	18 (14.1%)	60 (10.8%)	0.291
Mobility (active)	507 (74.2%)	83 (65.4%)	424 (76.3%)	0.011
Alcohol intake (g/day)	2.2 (0.3–11.9)	1.8 (0.4–7.8)	2.4 (0.3–13.1)	0.527
Calcium intake (mg/day)	824 (637–1,037)	821 (597–1,089)	826 (640–1,032)	0.901
Mood disorder (lifetime)	255 (36.6%)	78 (59.4%)	177 (31.3%)	<0.001
Physical multimorbidity (lifetime)	115 (16.5%)	29 (22.1%)	86 (15.2%)	0.055
Socioeconomic status				0.637
Quintile 1 (lowest)	103 (14.8%)	23 (17.6%)	80 (14.2%)	
Quintile 2	76 (10.9%)	16 (12.2%)	60 (10.6%)	
Quintile 3	269 (38.7%)	46 (35.1%)	223 (39.5%)	
Quintile 4	135 (19.4%)	28 (21.4%)	107 (18.9%)	
Quintile 5	113 (16.2%)	18 (13.7%)	95 (16.8%)	
Medication use (current)				
Antiresorptive agents	21 (3.0%)	3 (2.3%)	18 (3.2%)	0.589
Glucocorticoids	20 (2.9%)	4 (3.1%)	16 (2.8%)	0.778
Hormone therapy	26 (3.7%)	3 (2.3%)	23 (4.1%)	0.333
Antidepressants	114 (16.4%)	32 (24.4%)	82 (14.5%)	0.006
Unadjusted BMD (g/cm^2^)				
Spine	1.20 ± 0.18	1.22 ± 0.18	1.20 ± 0.18	0.306
Hip	0.92 ± 0.14	0.94 ± 0.14	0.92 ± 0.14	0.048
Total body	1.14 ± 0.11	1.16 ± 0.11	1.13 ± 0.11	0.055
Osteoporosis (current)	57 (8.2%)	8 (6.1%)	49 (8.7%)	0.335

*Values are given as median (interquartile range), mean ± standard deviation or n (%)*.

* *Comparing women with and without PD*.

*BMD, bone mineral density; PD, personality disorder*.

After age, weight adjustments, no differences were detected in spine, hip, or total body BMD between those with and without any PD (all *p* > 0.05).

The frequency of PD clusters was: 2.7%, cluster A (*n* = 19); <1%, cluster B (*n* = 5); and 15.4%, cluster C (*n* = 107).

Unadjusted and best models showing associations between PD clusters and BMD are presented in Table 2. Those with cluster A PD, compared to those without, had 6.7% lower hip BMD [age, weight adjusted mean 0.853 (95% CI 0.803–0.903) vs. 0.910 (95% CI 0.901–0.919) g/cm^2^, *p* = 0.027] and 3.4% lower total body BMD [age, weight, smoking, alcohol, calcium adjusted mean 1.102 (95% CI 1.064–1.140) vs. 1.139 (95% CI 1.128–1.150) g/cm^2^, *p* = 0.056]. No differences were detected in spine BMD between those with and without cluster A PD. Finally, no differences were detected in spine, hip, and total body BMD for those with and without cluster B or C PDs.

**Table 2:** Unadjusted and best models presenting associations between PD clusters and BMD using multiple regression.

**BMD site**	**PD status**	**Model I: unadjusted**			Model II: best model^*^		
		***B***	**SE**	***p***	***B***	**SE**	***p***
Spine	Cluster A	0.034	0.043	0.435	−0.010	0.039^b^	0.789
	Cluster B	0.026	0.083	0.756	−0.025	0.083^b^	0.761
	Cluster C	0.013	0.019	0.499	−0.005	0.018^b^	0.769
Hip	Cluster A	−0.007	0.033	0.834	−0.057	0.026^a^	0.027
	Cluster B	0.006	0.064	0.922	−0.023	0.050^a^	0.645
	Cluster C	0.034	0.015	0.027	0.009	0.012^a^	0.428
Total body	Cluster A	0.009	0.025	0.724	−0.037	0.019^c^	0.056
	Cluster B	−0.021	0.049	0.675	−0.052	0.041^d^	0.202
	Cluster C	0.023	0.011	0.045	0.005	0.009^e^	0.599

* Best models, adjusted for

a age and weight;

b age, weight, and alcohol;

c age, weight, smoking, alcohol, and calcium intake;

d age, weight, smoking, and alcohol;

e *age, weight, activity, and smoking*.

*B, regression coefficient; BMD, bone mineral density; SE, standard error; PD, personality disorder*.

## Discussion

To our knowledge, this study is the first to investigate bone health of women with PD in a population-based sample. It showed that those with cluster A PD have lower hip/total body BMD than those without. There were no associations between pooled PDs, cluster B or cluster C PD and BMD at any site.

Kahl et al. (7, 8) suggested that young women with borderline PD and current major depression may be a group at risk of developing osteoporosis. In their clinical study, the BMD of 24 women with MDD, 16 with borderline PD, 23 with MDD and comorbid borderline PD, and 20 healthy control participants were compared (7). The MDD only group were further divided into younger/older age groups [mean age 30 years (MDD30); 43 years (MDD43), respectively]. Women with MDD and comorbid borderline PD had significantly lower BMD at the lumbar spine compared with women with borderline PD alone and the younger women with MDD (MDD30) (7). No significant associations were reported between the groups at the other sites (i.e., right and left femur and forearm of non-dominant hand) (7). Markers of bone turnover were also measured, and compared with healthy control participants, elevated levels of osteocalcin were reported among women with MDD and borderline PD group (7). However, C-terminal degradation products of type I collagen (CTx) were highest among the MDD43 group, and tumor necrosis factor-α, interleukin-6, and osteoprotegerin were highest among the MDD30 group (7). To summarize, they postulated that rather than the presence of borderline PD alone, the acuteness of a co-occurring MDD may contribute to bone loss through associated immune/endocrine disturbances (7, 8). Though not directly comparable, we detected no direct association between cluster B and BMD. However, the lack of associations between cluster B PD and BMD detected in the present study could also be due to power constraints.

The novelty of the current research precludes meaningful comparisons with other studies on cluster A/C in relation to bone health. It is possible that psychological/social factors/processes that are characteristic of cluster A PDs, including enduring social isolation and avoidance coping, may be a barrier to engaging with healthcare providers for preventative, ongoing monitoring of physical health and/or engagement with treatments (2, 19)—including bone health. It is also plausible that vitamin D intake could be a biological mechanism underlying the associations.

In terms of study limitations, power limitations prevented analyses investigating specific PDs, other psychiatric disorders, and underlying biological mechanisms. Specifically, we did not collect and, therefore, did not examine the role of vitamin D, which is a known regulator of bone and mineral metabolism, as a confounder/moderator of associations between PD and BMD. This may be an area that warrants further research, given the suggestion that vitamin D may play a role in the manifestation of reduced quality of life *via* poorer health status among postmenopausal women referred for evaluation for osteopenia/osteoporosis (20) and, separately, with poorer psychological and somatic symptomatology (21). We also acknowledge that the classification of PD is undergoing significant reform, and dimensional measures of personality were not examined. Strengths of this study include the wide adult age range, gold standard tool for assessing psychiatric disorders, and adjustment for many known confounders.

Our preliminary study supports an association between PD and bone health. A more substantive evidence base is needed to further uncover the clinical implications of a relationship between PD and bone health. It may be important to determine whether monitoring the bone health of people with PD and comorbidities is warranted, given polypharmacotherapy is often prescribed for the treatment of PD (22), and specific agents, such as selective serotonin reuptake inhibitors, anticonvulsants, and antipsychotics, are associated with decreased bone mass and increased risk for osteoporosis and related fractures (23). This may require a coordinated effort between primary and endocrinological care, given indications that psychological/behavioral symptoms of PD may be a barrier to general health treatment/compliance for some individuals (4).

Given the paucity of literature, replication and longitudinal research including the examination of underlying mechanisms and sex differences are warranted.

## Data Availability Statement

The raw data supporting the conclusions of this article will be made available by the authors, without undue reservation.

## Ethics Statement

The studies involving human participants were reviewed and approved by Barwon Health Human Research Ethics Committee. The patients/participants provided their written informed consent to participate in this study.

## Author Contributions

LW conceived and designed the study. LW and AS performed the analyses. All authors contributed to the interpretation of the data and drafting of the article, provided critical revisions to the article, and approved the final version to be published.

## Conflict of Interest

The authors declare that the research was conducted in the absence of any commercial or financial relationships that could be construed as a potential conflict of interest.

## Abbreviations

BMD: bone mineral density
CI: confidence interval
DSM: Diagnostic and Statistical Manual of Mental Disorders
IRSAD: Index of Relative Socio-economic Advantage and Disadvantage
MDD: major depressive disorder
PD: personality disorder
SCID-I/NP: The Structured Clinical Interview for Diagnostic and Statistical Manual of Mental Disorders Axis I Disorders, non-patient edition
SCID-II: The Structured Clinical Interview for DSM-IV Axis II personality disorders
SES: socioeconomic status.
